# A panel of kallikrein markers can predict outcome of prostate biopsy following clinical work-up: an independent validation study from the European Randomized Study of Prostate Cancer screening, France

**DOI:** 10.1186/1471-2407-10-635

**Published:** 2010-11-22

**Authors:** Amine Benchikh, Caroline Savage, Angel Cronin, Gilles Salama, Arnauld Villers, Hans Lilja, Andrew Vickers

**Affiliations:** 1 Hôpital Bichat-Claude Bernard, Paris, France; 2 Memorial Sloan Kettering Cancer Center, New York, NY, USA; 3 Centre Hospitalier Intercommunal Castres-Mazamet, Castres, France; 4 Hôpital Huriez, CHRU Lille, Lille, France

## Abstract

**Background:**

We have previously shown that a panel of kallikrein markers - total prostate-specific antigen (PSA), free PSA, intact PSA and human kallikrein-related peptidase 2 (hK2) - can predict the outcome of prostate biopsy in men with elevated PSA. Here we investigate the properties of our panel in men subject to clinical work-up before biopsy.

**Methods:**

We applied a previously published predictive model based on the kallikrein panel to 262 men undergoing prostate biopsy following an elevated PSA (≥ 3 ng/ml) and further clinical work-up during the European Randomized Study of Prostate Cancer screening, France. The predictive accuracy of the model was compared to a "base" model of PSA, age and digital rectal exam (DRE).

**Results:**

83 (32%) men had prostate cancer on biopsy of whom 45 (54%) had high grade disease (Gleason score 7 or higher). Our model had significantly higher accuracy than the base model in predicting cancer (area-under-the-curve [AUC] improved from 0.63 to 0.78) or high-grade cancer (AUC increased from 0.77 to 0.87). Using a decision rule to biopsy those with a 20% or higher risk of cancer from the model would reduce the number of biopsies by nearly half. For every 1000 men with elevated PSA and clinical indication for biopsy, the model would recommend against biopsy in 61 men with cancer, the majority (≈80%) of whom would have low stage *and *low grade disease at diagnosis.

**Conclusions:**

In this independent validation study, the model was highly predictive of prostate cancer in men for whom the decision to biopsy is based on both elevated PSA and clinical work-up. Use of this model would reduce a large number of biopsies while missing few cancers.

## Background

Prostate specific antigen (PSA) is the only molecular marker routinely used for the early detection of a common cancer. Data from the 2001 US Behavioral Risk Factor Surveillance System are that 75% of men aged 50 years or older have had at least one PSA test and that, of men aged 50 to 69 years - the ages typically targeted in screening recommendations[1] - 54% reported having had a PSA test within the past year[2]. These numbers have remained fairly constant for data collected in 2002, 2004, and 2006[3]. Racial disparities in PSA testing have been described. African-Americans below 50 have higher rates of screening that younger White men and Hispanic men[4,5], likely due to explicit recommendations for an earlier start to screening in this population[1]. Older African-Americans and Hispanics have lower rates of screening than comparably aged White men, an effect largely attributable to differences in socio-economic status[3-5].

The recent results of two large, randomized trials give qualified support for the use of PSA screening. The value of PSA testing in men who would otherwise not be screened was assessed in the European Randomized Study of Prostate Cancer (ERSPC). A total of 182,000 men in seven European countries were randomized to PSA screening or control. The background rate of PSA testing in these countries was low. At a median follow-up of nine years, PSA screening was associated with a statistically significant 20% relative reduction in the risk of prostate cancer death. This difference is likely to increase over time. However, this benefit came at high cost, with an estimated 48 men needing to be treated for prostate cancer in order to prevent one death, or two cases of metastasis, at 9 years [6]. The US-based PLCO trial, on the other hand, assessed a recommendation to screen in US men. As might be predicted from the population-based surveys described above, many of those accrued (~50%) had already had a PSA test. Moreover, many of the men randomized to the control group continued to have PSA tests irrespective of randomized assignment: 40% of men in the control group received a PSA test in the first year after randomization. At a median follow-up of 7 years, prostate cancer specific mortality was very low, with no difference between arms [7].

PSA is an imperfect marker of prostate cancer. Although highly specific to the prostate gland, PSA is not specific for prostate cancer. We have previously estimated that, each year, over 750,000 US men receive unnecessary prostate biopsy[8].

There is clearly a need for better markers. We have previously shown that a panel of four kallikrein markers - total PSA, free PSA, intact PSA and human kallikrein-related peptidase 2 (hK2) - is strongly predictive of prostate biopsy outcome. In our initial report[8], we calculated an area under the curve (AUC) of 0.83 for the kallikrein panel, compare to just 0.68 for a "base" model of total PSA and age alone. We reported that using the full kallikrein panel would reduce biopsy rates by more than 50% for men with elevated PSA while missing only a small number of cancers (31 out of 152 low-grade and 3 out of 40 high-grade cancers).

We subsequently validated these results in several independent cohorts of men. In the Rotterdam arm of the ERSPC, we found that the panel resulted in a similar improvement in predictive accuracy (AUC improved from 0.64 to 0.76) and reduction of biopsy rates (573 per 1000 men with elevated PSA) while missing only a small number of cancers (42 per 1000 men) [9]. These results have also been replicated in previously screened men [10,11].

In these prior studies, all men with an elevated PSA were referred for biopsy as per the ERSPC protocol. This is somewhat distinct to usual clinical practice in which men with elevated PSA are typically subject to clinical work-up before referral to biopsy. Clinical judgment takes into consideration patient's clinical history to rule out transient prostatic inflammation, to assess benign enlargement and evaluate prostate nodularity by digital rectal examination (DRE). Recommendation for biopsy might also take into consideration a range of other factors, such as prostate symptoms, history of benign prostate conditions, and family history of cancer. It is known that this type of clinical work-up can affect the properties of markers[12].

It is plausible that this type of clinical work-up and judgment would affect the properties of predictive models for prostate cancer. Here we aim to determine whether our previously created statistical model - developed on patients biopsied during the first round of the ERPSC-Rotterdam where almost all men with elevated PSA underwent biopsy - would retain its predictive value in men biopsied in ERSPC France, where biopsy following an elevated PSA was based on clinical judgment.

## Methods

### Patients

During 2001-2005, 11,395 men were randomized to receive screening as part of ERPSC-Tarn, France. Of these, 4,200 men agreed to participate. These rates of participation are lower than has been reported from other ERSPC sites. This is likely because France entered the study at a later time (2001 vs 1994 for the other centers) and PSA was already relatively common in France at that time, making subjects less likely to consent to randomization [13]. According to the ERSPC France protocol, the decision to biopsy was based on clinical judgment following additional work-up such as DRE or additional PSA test. If the repeat PSA was below 3 ng/ml, or the DRE was not suspicious, the urologist could advise against biopsy[14].

Laboratory methods were as for our prior publications [8,9]. Serum samples were retrieved from the archival serum bank in Tarn (where they had been stored frozen at -80°C after their initial processing within 3 hours from venipuncture) and shipped frozen on dry ice to Memorial Sloan-Kettering Cancer Center in 2008 for the analysis of hK2. Samples were then shipped to the Wallenberg Research Laboratories, Department of Laboratory Medicine, Lund University, University Hospital in Malmö, Sweden in 2009 for analysis of free, total and intact PSA. Free and total PSA were measured using the dual-label DELFIA Prostatus^® ^total/free PSA Assay (Perkin-Elmer, Turku, Finland). Intact PSA and hK2 were measured by using F(ab')2 fragments of the monoclonal capture antibodies in order to significantly reduce the frequency of non-specific assay interference. The intact PSA assay measures only free, uncomplexed intact PSA (i.e. not cleaved at Lys145-Lys146). All analyses were conducted blind to biopsy result.

#### Statistical Methods

Our aim in this paper was to independently validate the models built using participants of the Rotterdam arm of the ERSPC. The development of these models has been described previously[9]. In brief, we created a "base" model using data routinely available in current clinical practice (age, PSA, DRE) and a "full" model also incorporating levels of total PSA, free PSA, intact PSA and hK2. In the original model, all markers were entered as restricted cubic splines with knots at the tertiles to allow a non-linear relationship with outcome. Multivariable logistic regression was used to fit all models.

We made several modifications to simplify our model after completion of our research on the Rotterdam cohort but before it was applied to the ERSPC Tarn data. In brief, we eliminated non-linear terms for iPSA and hK2 on the grounds that they substantially increased model complexity yet, when evaluated on the training set, did not markedly improve predictive accuracy. We have previously published an evaluation of the use of this model on previously screened men [15]. Therefore the Tarn data was used for an entirely independent replication of our prediction model.

We compared the value of the kallikrein models to a base model of established predictors: age, total PSA and DRE result. Predictive accuracy was reported as the area under the receiver operating characteristics curve (AUC). Confidence intervals and inference statistics for differences between AUCs were obtained using the method of Delong[16]. Confidence intervals for the differences in AUC between models were calculated by bootstrap methods. High grade cancer was defined as Gleason grade 7 or higher. The AUC for high grade cancer was calculated from the predicted probabilities of any cancer, that is, we did not build a separate model for the outcome of high grade disease. For these analyses, patients with low-grade cancer were classified the same as patients with negative biopsy when high-grade cancer was the outcome: five patients with missing information on grade were considered to have low-grade cancer.

To evaluate the clinical implications of these models, we used decision curve analysis [17]. This method estimates the "net benefit" of using a prediction model by summing the benefits (true positives) and subtracting the harms (false positives), where the latter is weighted by a factor related to the relative harm of a missed cancer compared to an unnecessary biopsy. The weighting is derived from the probability of prostate cancer at which a patient would choose to be biopsied. As this threshold probability can vary from patient to patient, net benefit is calculated across a range of probabilities; as in previous papers, we chose 10% - 40% as a reasonable range. Five patients missing Gleason grade were excluded from the analyses of high grade disease. Statistical analyses were conducted using Stata 11.0 (StataCorp LP, College Station TX).

## Results

In total, 629 men had an elevated PSA (>= 3 ng/ml) in round 1. Of these, 370 (59%) men received a biopsy. Table 1 summarizes the additional workup (PSA test and DRE) that men received, separately for those who did and did not receive a biopsy. Overall, 489 (78%) men received some form of further workup, either a second PSA test (n = 123; 20%) or a DRE (n = 447; 71%). Men who refused biopsy were more likely to have a subsequent PSA test than those who received a biopsy (28% vs 14%), but were less likely to have had an abnormal DRE (44% vs. 90%).

**Table 1:** Details of further diagnostic workup of men with an elevated PSA.

	Men not undergoing subsequent biopsy N = 259	Men who received a biopsy N = 370
*Repeat PSA*	72 (28%)	51 (14%)

*PSA*	3.1 (2.4, 3.6)	4.6 (3.6, 7.2)

*PSA < 3 ng/ml (% of those with second PSA)*	34 (47%)	5 (10%)

*Digital rectal exam*	114 (44%)	333 (90%)

*Abnormal findings (% of those with DRE)*	16 (14%)	115 (35%)

Table 2 shows the characteristics of the sample. Men who were not recommended for biopsy had slightly lower PSA than those who were biopsied. For some patients (n = 108) who underwent a biopsy, clinical data or blood samples were not available. There were no important differences between biopsied men with and without marker data available: the cancer rate is 32% vs. 30% respectively, with 54% and 44% having high-grade disease (i.e. Gleason ≥7 on biopsy). The majority of men with complete data received an extended biopsy scheme; 45 (17%) received a biopsy with fewer than 10 cores.

**Table 2:** Patient characteristics.

	Men with an elevated PSA not undergoing subsequent biopsy N = 259	Men who received a biopsy N = 370			
		
		Complete marker and DRE data N = 262		Missing marker or DRE N = 108	
		
		No Cancer N = 179	Cancer N = 83	No Cancer N = 76	Cancer N = 32
Age at screening (years)	64 (59, 67)	63 (59, 67)	65 (61, 69)	*65 (60, 67)*	*65 (61, 68)*

Total PSA	3.75 (3.12, 4.80) N = 228	4.23 (3.36, 5.58)	4.86 (3.81, 7.23)	-	-

Free PSA	0.88 (0.67, 1.15) N = 228	1.01 (0.77, 1.32)	0.96 (0.67, 1.36)	-	-

Intact PSA	0.38 (0.27, 0.56) N = 228	0.45 (0.34, 0.63)	0.50 (0.33, 0.75)	-	-

Human Kallikrein 2	0.061 (0.038, 0.094) N = 216	0.061 (0.036, 0.090)	0.088 (0.050, 0.132)	-	-

Number of biopsy cores	-	12 (10, 12)	12 (10, 12)	*11 (6, 12)*	*10 (6, 12)*

Clinical T Stage					

T1	-	-	31 (37%)	-	*13 (41%)*

T2	-	-	39 (47%)	-	*12 (38%)*

T3	-	-	8 (10%)	-	*1 (3%)*

*Missing*	-	-	5 (6%)	-	*6 (19%)*

Biopsy Gleason Grade					

<= 6	-	-	33 (40%)	-	*17 (53%)*

7	-	-	33 (40%)	-	*11 (34%)*

>= 8	-	-	12 (14%)	-	*3 (9%)*

*Missing*	-	-	5 (6%)	-	*1 (3%)*

All values are median (interquartile range) or frequency (proportion).

The predictive accuracy of the model built on Rotterdam participants and applied to Tarn individuals is shown in Table 3. The full-kallikrein panel had significantly higher predictive accuracy than the base model consisting of the established predictors of PSA, age and DRE result alone: the AUC improved from 0.628 to 0.782 (improvement of 0.154; 95% CI: 0.086, 0.216). Similar improvements were seen for the outcome of high-grade cancer (Table 3).

**Table 3:** Predictive accuracy of models built on Rotterdam participants when applied to Tarn participants.

	Clinical Model	
	**Any cancer**	**High grade cancer**

Base model	0.628 (0.552, 0.704)	0.767 (0.687, 0.847)

Full kallikrein panel	0.782 (0.719, 0.845)	0.870 (0.807, 0.933)

*without *iPSA	0.753 (0.687, 0.818)	0.842 (0.776, 0.907)

*without *fPSA	0.688 (0.619, 0.758)	0.795 (0.721, 0.868)

*without *hK2	0.770 (0.706, 0.833)	0.853 (0.786, 0.919)

Cancers with pathologic Gleason score 7 or higher were considered high grade. Base model includes age and total PSA (with splines) and DRE result. Full model includes age, total PSA (with splines), free PSA (with splines), intact PSA, hK2 and DRE result.

To evaluate the individual contribution of each kallikrein, we fit a model to the initial Rotterdam training set and evaluated it on the Tarn cohort, iteratively removing each marker. Free PSA appeared to have the largest contribution but removing intact PSA and hK2 from the model also led to a reduction in AUC. This supports the use of all four kallikreins in the marker panel.

Table 4 illustrates the clinical implications of using the kallikrein model to determine biopsy in men with elevated PSA and considered eligible for biopsy after clinical work-up. Using a threshold of 20% or greater risk from the model as the indication for biopsy would lead to a near halving of the biopsy rate. For every 1000 men with elevated PSA and clinical indication for biopsy, 492 would avoid biopsy by use of the model; this would come at the expense of 61 men with cancer being advised against biopsy. The majority of these cancers would be low stage (all would be T1 or T2) and low grade (only 12 would be Gleason 7 or higher).

**Table 4:** Reduction in biopsies/cancers detected using as a threshold for biopsy a 20% or higher probability of cancer.

	No. biopsies		**No. cancers**:		**No. high grade cancers**:	
	**Performed**	**Avoided**	**Found**	**Missed**	**Found**	**Missed**

**Biopsy all men at risk**	1000	0 (0%)	317	0 (0%)	175	0 (0%)

**Biopsy if >= 20% risk from full model**	508	492 (49%)	256	61 (19%)	163	12 (4%)

Numbers are given per 1000 men with elevated PSA (>= 3 ng/ml) and recommended for biopsy after clinical work-up.

**Figure 1:**
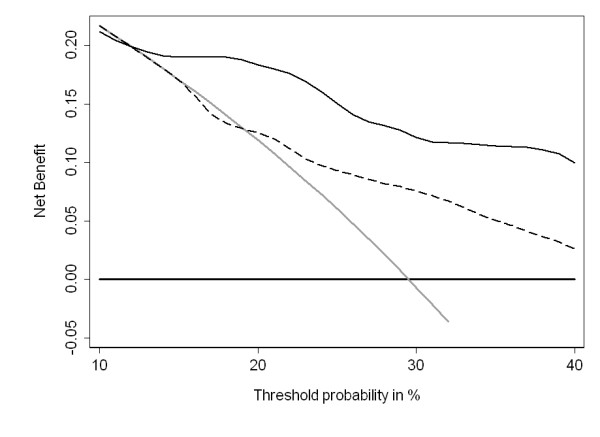
**Decision curve analysis**. The dashed line is for the base model (age, DRE result, and total PSA); the solid line is for the full model (age, DRE result, total PSA, free PSA, intact PSA, and hK2). As a comparison, the thin grey line is for the strategy of biopsying all men and the thick black line for biopsying no man.

To evaluate further the clinical implications of using the four kallikrein panel, we created decision curves for the outcome of any prostate cancer diagnosis (Figure 1). The net benefit of the four kallikrein panel was superior to the base model and a "biopsy all" strategy for all threshold probabilities above 12%. Thus use of the panel would be clinically beneficial for all but the most risk averse men.

We have previously reported that the predictive accuracy of the full kallikrein panel is lower among men with a history of PSA screening[10,11]. This finding is largely due to the dramatically reduced predictive value of PSA in these men. We planned a secondary analysis stratifying by whether or not men reported a history of PSA testing. Approximately half the men (n = 133; 51%) reported a history of PSA testing. The results from the stratified analysis confirmed both the main findings reported here and our previous results that the accuracy is reduced in previously screened men. Use of the kallikrein panel had greater discrimination that the base model for both previously screened men (AUC 0.552 vs. 0.679) and those without a history of PSA screening (0.692 vs. 0.865).

## Discussion

We have replicated our previously published finding that a panel of four kallikreins can predict the result of biopsy for prostate cancer in men with elevated PSA. Critically, we have shown that the model retains its value in men who were clinically evaluated before an extended biopsy. Use of the panel would dramatically reduce biopsy rates while missing relatively few cancers, most of which are low grade, limited stage prostate cancers typically thought to constitute overdiagnosis.

The aim of clinical work-up is to distinguish benign from malignant causes of PSA elevation. For example, of men with a second PSA lower than 3 ng/ml only a minority (13%) went forward to biopsy; in comparison, 88% of those with a positive DRE were biopsied. In total, 40% of men with elevated PSA were considered insufficiently high-risk after work-up to warrant biopsy. It is plausible that aspects of benign and malignant prostate disease captured by our panel would overlap with those detected clinical work-up. As such, it seemed possible that the four kallikrein panel's contribution to risk-stratification would be limited in the presence of clinical judgment. Yet our findings indicate that the kallikrein panel significantly improves prediction and would lead to improved referral to biopsy, providing strong support for the use of the full kallikrein panel in clinical practice.

Other promising markers of prostate cancer, such as PCA3, have also been shown to enhance the discrimination of prostate cancer on biopsy[18,19]; however, the improvements are smaller than those from the full kallikrein panel. For example, Deras et. al. reported that addition of PCA3 to a model including prostate volume, DRE result and PSA improved the discrimination of prostate cancer on biopsy from and AUC of 0.67 to 0.75[20]. In comparison, we show here that use of the full kallikrein panel would increase the AUC of prostate cancer from 0.63 to 0.78 over that of age, PSA and DRE result alone.

A major strength of this paper is the close concordance between our prior results and those reported here. We found basing biopsy decisions on the kallikrein panel would lead to 492 fewer biopsies per 1000 men with an elevated PSA, but would miss 61 men with cancer of whom 12 had high-grade disease. The comparable figures in the Rotterdam cohort were 513, 66 and 12. Of note is the fact that the incidence of prostate cancer is higher in Tarn than in Rotterdam (317 versus 277 cancers found per 1000 men with an elevated PSA) - clear evidence that clinical judgment was able to select men who were at higher risk of cancer. Yet despite the higher incidence of prostate cancer in Tarn, use of the four kallikrein panel in this cohort did not lead to a greater number of missed cancers.

There are several possible limitations of this study. First, we do not know whether all men who refused biopsy did not in fact have cancer. However, our study is not subject to verification bias [21], as we only analyzed men who underwent biopsy. Indeed, we see it as a positive advantage of our study that not all men with elevated PSA underwent biopsy, as this reflects usual clinical care. Second, the Tarn arm of the ERSPC had a much lower rate of participation than the other arms of the ERSPC and may not represent a population-based cohort of men. Nonetheless, our prior studies evaluated the kallikrein panel in representative population-based cohorts and found consistent results to those reported here.

## Conclusions

We have independently replicated our prior finding that a previously developed statistical model, based on four kallikreins, is a strong predictor of biopsy outcome in men with elevated PSA deemed eligible for biopsy after clinical work-up. Using a decision analytic approach, we have also demonstrated that use of the model can importantly reduce biopsy rates while delaying the diagnosis of only a limited number of cancers, the majority of which are of low grade and low stage. This suggests that use of the panel to determine biopsy in routine clinical practice would improve decision making about biopsy.

## Abbreviations

PSA: prostate-specific antigen; AUC: area under the receiver operating characteristic curve; hK2: human kallikrein-related peptidase 2; DRE: digital rectal exam; ERSPC: European Randomised study of Screening for Prostate Cancer

## Competing interests

Dr. Hans Lilja holds patents for free PSA and hK2 assays and is named as co-inventor on a patent application for intact/nicked PSA assays.

## Authors' contributions

AB, GS and A. Villers participated in the design of the study and coordinated collection of patient data. AV carried out the biomarker assays. CS and A. Vickers performed the statistical analysis and drafted the manuscript. AV and HL conceived of the study, and participated in its design and coordination. All authors read and approved the final manuscript.

## Pre-publication history

The pre-publication history for this paper can be accessed here:

http://www.biomedcentral.com/1471-2407/10/635/prepub
